# Impact of high-altitude hypoxia on *Helicobacter pylori*-induced gastritis pathological manifestations and inflammatory responses

**DOI:** 10.1186/s40101-024-00364-5

**Published:** 2024-07-05

**Authors:** Chunxia Li, Xuehong Wang, Sen Cui

**Affiliations:** 1 Clinical Medical College of Qinghai University, Xining, Qinghai Province China; 2 Department of Gastroenterology, Qinghai University Affiliated Hospital, Xining, Qinghai Province China; 3 Department of Hematology, Qinghai University Affiliated Hospital, 29 Tongren Road, Xining, Qinghai Province 810001 China

**Keywords:** High altitude, Hypoxia, *Helicobacter pylori*, Chronic gastritis

## Abstract

**Background:**

Chronic gastritis caused by *Helicobacter pylori* (Hp) infection is a common gastrointestinal disorder. Despite the high prevalence of Hp infection and chronic gastritis in the Tibetan Plateau, there is a lack of studies elucidating the influence of plateau hypoxia on Hp-induced gastritis. This study aimed to investigate the impact of high-altitude hypoxia on Hp-induced gastritis, particularly focusing on pathological manifestations and inflammatory responses.

**Methods:**

This study was conducted from July 2023 to March 2024 at the Department of Gastroenterology, Affiliated Hospital of Qinghai University. Ninety patients diagnosed with chronic gastritis were enrolled in the study and divided into four groups based on their residential altitude and Hp infection status. Data on endoscopic and pathological characteristics were collected, along with serum oxidative stress and inflammatory markers.

**Results:**

Patients with Hp gastritis exhibit distinctive features in the gastric mucosa, including diffuse erythema, enlarged folds, and white turbid mucus during endoscopy. Notably, individuals with Hp gastritis at high altitudes show a higher prevalence of diffuse erythema and enlarged folds. Pathological analysis reveals that these patients have elevated gastric mucosal inflammation scores and increased chronic and active inflammation. Furthermore, individuals with Hp gastritis at high altitudes demonstrate elevated levels of serum TNF-α, IL-1β, IL-6, and MDA, as well as reduced serum SOD and GSH-Px activities.

**Conclusions:**

High-altitude hypoxia may exacerbate gastric mucosal damage by enhancing oxidative stress and inflammatory response induced by Hp infection.

## Introduction

*Helicobacter pylori* (Hp) is a gram-negative, microaerobic spirochete that colonizes and persists in the human gastric mucosa [1]. Hp infections are linked to gastroduodenal disorders such as chronic gastritis, peptic ulcers, and gastric malignancies, as well as extragastric conditions including iron deficiency anemia, primary immune thrombocytopenia, and vitamin B12 deficiency [2]. The estimated global prevalence of Hp infection decreased from 58.2% in the 1980–1990 period to 43.1% in the 2011–2022 period [3], with China maintaining a high prevalence rate of 44.2% [4]. Chronic gastritis, attributed mainly to Hp infection, is the predominant digestive ailment in China [5]. Research indicates that nearly all individuals infected with Hp progress to chronic active gastritis [6], with some developing gastric mucosal atrophy and/or intestinal metaplasia following the Correa pattern, leading to gastric cancer [7]. This progression has a significant impact on the quality of life and overall health of individuals.

The pathogenesis of gastritis induced by Hp infection primarily involves Hp adhesion and colonization, release of virulence factors, and the initiation of oxidative stress and immune-inflammatory responses [8]. Environmental factors, alongside Hp and host factors, significantly contribute to the onset and progression of this condition [9]. The Tibetan Plateau, mainly encompassing Tibet and Qinghai, stands as China’s largest and the world’s highest plateau, characterized by low pressure and oxygen levels. Prolonged exposure of plateau inhabitants to these unique conditions leads to various pathophysiological changes across multiple physiological systems, directly impacting overall organism function. According to the Sixth National Health Service Survey, conducted in 2018, the top five prevalent diseases in Tibet are digestive system disorders, cardiovascular ailments, musculoskeletal conditions, respiratory issues, and genitourinary illnesses, with digestive disorders showing the highest prevalence [10]. Despite the high prevalence of Hp infection and chronic gastritis in the Tibetan Plateau, there is a lack of studies elucidating the influence of plateau hypoxia on Hp-induced gastritis. This study aims to investigate the impact of plateau hypoxia on oxidative stress and inflammatory response of the gastric mucosa in patients with Hp gastritis in the Tibetan Plateau region. The findings aim to establish an experimental foundation for understanding the pathogenesis of Hp gastritis in high-altitude regions.

## Methods

### Study population and location

A total of 100 with chronic gastritis were recruited from the Department of Gastroenterology at the Affiliated Hospital of Qinghai University between July 2023 and March 2024. The patients were categorized into four groups based on their residential altitude and Hp infection: Hp-negative chronic gastritis group (Hp^−^ X group, *n* = 26) and Hp-positive chronic gastritis group (Hp^+^ X group, *n* = 25) residing in the urban area of Xining City, Qinghai Province (average elevation of approximately 2261 m); as well as Hp-negative chronic gastritis group (Hp^−^ G group, *n* = 22) and Hp-positive chronic gastritis group (Hp^+^ G group, *n* = 27) living at an elevation of ≥ 3500 m in Qinghai.

### Inclusion criteria

We included patients between 18 and 65 years old who had lived in Qinghai for their whole lifetime and whose endoscopic examination was consistent with chronic gastritis with reference to the Chinese Guidelines for the Diagnosis and Treatment of Chronic Gastritis (2022, Shanghai) [5].

### Exclusion criteria

Patients were excluded if they had received proton pump inhibitors (PPIs), H2-receptor antagonists (H2RAs), potassium-competitive acid blockers (P-CABs), antibiotics, bismuth, micro-ecological agents, or traditional Chinese medicines with antibacterial effects within the previous 1 month. Patients with severe heterotrophic hyperplasia, upper gastrointestinal hemorrhage, peptic ulcers, malignant tumors, or gastric surgeries were also excluded, as well as those with a combination of cardiac, hepatic, or renal failure, hypertension, diabetes mellitus, thyroid diseases, or autoimmune disorders. Additionally, pregnant and breastfeeding women, or those with serious bad habits such as smoking or alcoholism, were also excluded.

### Endoscopy and mucosal biopsy samples

Endoscopy was conducted on the patient in a fasting state, revealing various pathological changes in the gastric mucosa, including mucosal punctate flaky erythema, diffuse erythema, hemorrhagic dots or plaques, enlarged folds, white turbid mucus, and mucosal erosion. Two pieces of gastric mucosal tissue were taken from the antrum near the lesser curvature, 2–3 cm from the pylorus.

### Detection of Hp

The presence of Hp infection was confirmed through the use of ^13^C-urea breath test and gastric mucosal immunohistochemistry. A positive outcome in either of these tests indicates an Hp infection, while a negative result in both tests rules out the presence of an Hp infection.

### Histological assessment

The gastric tissues were fixed in 4% paraformaldehyde, dehydrated with alcohol, routinely embedded in paraffin, cut at 4 µm and stained with hematoxylin and eosin (H&E) for histopathology. Gastric histology was evaluated by 1 pathologist who was blinded to the other assays. Polymorphonuclear neutrophils infiltration, which indicates active gastric inflammation, and mononuclear inflammatory cells infiltration, which indicates chronic gastric inflammation, were assessed on a 4-grade scale (0, 1, 2, and 3, corresponding to none, mild, moderate, and severe, respectively), according to the Updated Sydney System [11] and Chinese Guidelines for the Diagnosis and Treatment of Chronic Gastritis [5] (Table 1).

**Table 1:** Grading criteria for chronic inflammation and activity in gastric mucosa

Grade	Chronic inflammation	Activity
0	The presence of fewer than five mononuclear inflammatory cells per high-power field	Absence of neutrophil infiltration in the context of chronic inflammation
1	Mononuclear inflammatory cells are sparse and confined to the superficial layer of the mucosa, not exceeding one-third of the mucosal layer	The mucosal lamina propria exhibits infiltration of a small number of neutrophils
2	Mononuclear inflammatory cells are more densely populated, not exceeding two-thirds of the mucosal layer	Neutrophils are predominantly found in the mucosal layer and can be observed within surface epithelial cells, crypt epithelial cells, or glandular epithelium
3	Mononuclear inflammatory cells are densely populated throughout the mucosal layer	Neutrophils are more densely packed, and in addition to moderate observations, small abscesses may also be present

### Evaluation of oxidative stress and inflammation response

Blood samples were collected into serum separator tubes, and serum was prepared by centrifuging (3000 r/min, 15 min) at 4 ℃. Commercial kits obtained from Wuhan Abbkine Technology Co., Ltd. in China were utilized for the evaluation of tumor necrosis factor-α (TNF-α), Interleukin (IL)-1β, and IL-6, malondialdehyde (MDA), superoxide dismutase (SOD), and glutathione peroxidase (GSH-Px) levels in serum.

### Statistical analysis

Data analysis and statistical graph generation were conducted using SPSS 27.0 and GraphPad Prism 9.5.1. The statistical differences between groups were determined by chi-square test or Kruskal–Wallis test followed by the Bonferroni correction post-hoc test, or one-way ANOVA followed by the least significant difference (LSD) post-hoc test, or Kruskal–Wallis test followed by Dunnett’s T3 post-hoc test. A *p*-value of less than 0.05 was considered statistically significant.

#### Ethics

This study was ethically approved by the Medical Ethics Committee of Qinghai University Affiliated Hospital (P-SL-2023–456). Prior to participation, all individuals provided informed consent after being fully briefed on the study and their rights.

## Results

### There was no statistically significant difference between the pairs of groups in the mean age or gender of patients (Table 2)

**Table 2:** General information of subjects participating in the study

Groups	*n*	Gender		Age(x̅ ± s, year)
		Male	Female	
Hp^−^ X	26	11	15	48.23 ± 10.01
Hp^−^ G	22	9	13	46.23 ± 10.61
Hp^+^ X	25	12	13	47.48 ± 10.45
Hp^+^ G	27	17	10	43.26 ± 11.00

#### Endoscopic observation of pathological changes in gastric mucosa

The study findings (Table 3) revealed a significantly higher prevalence of diffuse erythema, enlarged folds, and white turbid mucus in the gastric mucosa of the Hp^+^ X group compared to the Hp^−^ X group (*p* < 0.05). However, there were no statistically significant differences in the proportions of punctate erythema, hemorrhagic dots or plaques, and mucosal erosions between the Hp^+^ X and Hp^−^ X groups (*p* ≥ 0.05). The Hp^+^ G group exhibited a significantly higher proportion of gastric mucosa with diffuse erythema, enlarged folds, and white turbid mucus compared to the Hp^−^ G group (*p* < 0.05). Conversely, the proportions of punctate erythema, hemorrhagic dots or plaques, and mucosal erosion did not differ significantly between the Hp^+^ G and Hp^−^ G groups (*p* ≥ 0.05). Furthermore, patients in the Hp^+^ G group showed a notably higher prevalence of diffuse erythema and fold enlargement than those in the Hp^+^ X group (*p* < 0.05). These findings suggest that patients with Hp gastritis typically present with gastric mucosa characterized by diffuse erythema, fold enlargement, and white turbid mucus during endoscopy. Additionally, the prevalence of diffuse erythema and enlarged folds in the gastric mucosa appears to be elevated in patients with Hp gastritis in low-oxygen plateau environments.

**Table 3:** Endoscopic observation of pathological changes in gastric mucosa

Characteristic	Hp^−^ X(*n* = 26)	Hp^−^ G(*n* = 22)	Hp^+^ X(*n* = 25)	Hp^+^ G(*n* = 27)
Punctate erythema	15 (57.7%)	13 (59.1%)	15 (60.0%)	7 (25.9%)
Diffuse erythema	0 (0.0%)	1 (4.5%) ^#^	8 (32.0%) ^*#^	20 (74.1%)
Enlarged folds	0 (0.0%)	3 (13.6%) ^#^	8 (32.0%) ^*#^	20 (74.1%)
White turbid mucus	1 (3.8%)	2 (9.1%) ^#^	10 (40.0%) ^*^	13 (48.1%)
Hemorrhagic dots or plaques	2 (7.7%)	5 (22.7%)	4 (16.0%)	9 (33.3%)
Mucosal erosion	10 (38.5%)	13 (59.1%)	16 (64.0%)	20 (74.1%)

Note: Data were analyzed by chi-square test followed by Bonferroni’s correction post-hoc test

^*^p < 0.05 VS. Hp^−^ X group; ^#^*p* < 0.05 VS. Hp^+^ G group

### Histopathological evaluation of gastric tissue

#### *Pathological changes in the gastric mucosa of patients in each group were observed using HE staining (*Fig. 1A*)*

**Fig. 1:**
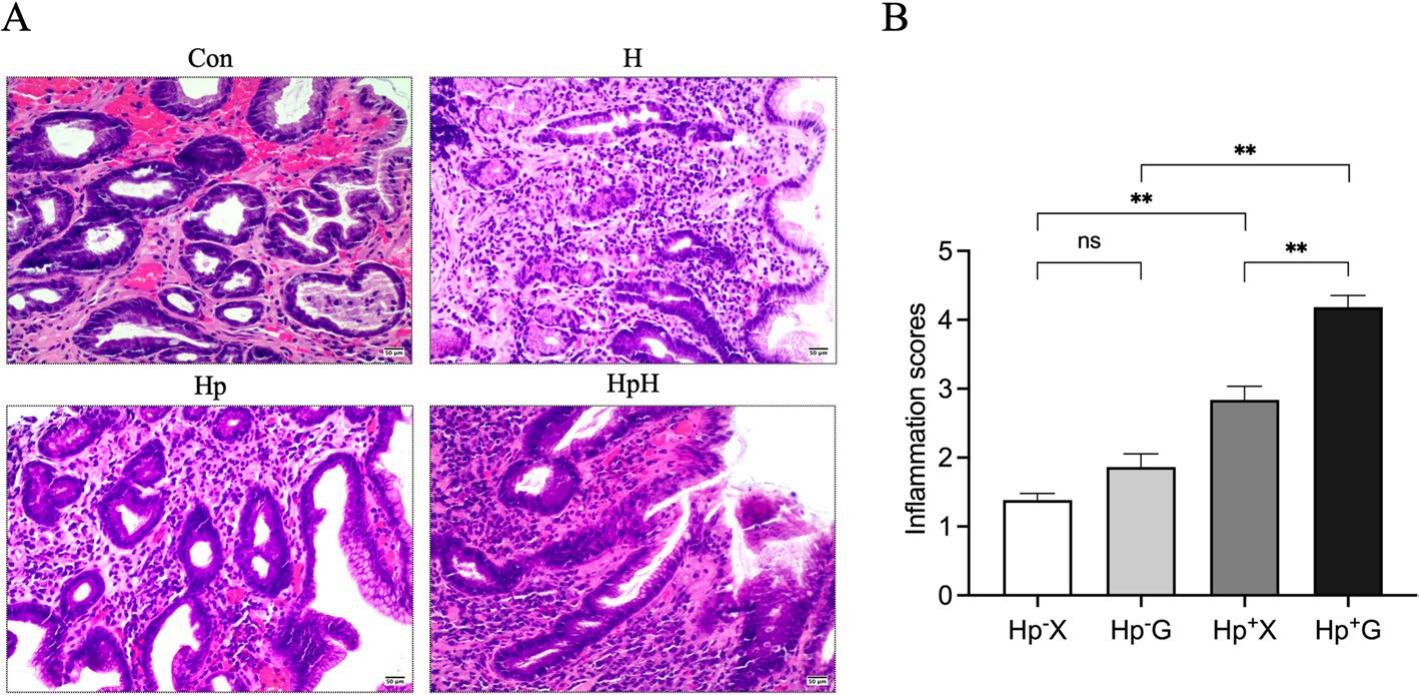
**A** HE staining of the gastric tissues (× 200). Scale bar: 50 µm. **B** Inflammatory scores of gastric tissue sections in each group. Data were represented as mean ± SEM and analyzed by Kruskal–Wallis test followed by Dunnett’s T3 post-hoc test. ^**^p < 0.01, ns, not significant

Patients in the Hp^−^ X group and Hp^−^ G group exhibit gastric mucosal changes characterized by partial necrosis and shedding of epithelial cells, slight disorganization of glandular arrangement, and mild inflammatory cell infiltration. In contrast, patients in the Hp^+^ X group show varying degrees of congestion and swelling, along with epithelial cell necrosis and shedding, disordered glandular arrangement, and significant infiltration of inflammatory cells in the lamina propria. Patients in the Hp^+^ G group display similar gastric mucosal pathology as the Hp^+^ X group but with more severe manifestations. The scores of neutrophil infiltration and monocyte infiltration were combined to assess gastric mucosal inflammation (Fig. 1B). The results suggest that the patients in the Hp^+^ G group exhibited the highest gastric mucosal inflammation score among the four groups, followed by those in the Hp^+^ X group. There was no statistically significant difference in gastric mucosal inflammation scores between patients in the Hp^−^ X group and the Hp^−^ G group.

#### The grading of chronic inflammation in the gastric mucosa of patients in each group revealed significant differences (Table 4)

**Table 4:** Grading of chronic inflammation of the gastric mucosa

Groups	*n*	Chronic gastritis severity			
		0	1	2	3
Hp^−^ X	26	0 (0.0%)	20 (76.9%)	6 (23.1%)	0 (0.0%)
Hp^−^ G	22	0 (0.0%)	12 (54.5%) ^#^	10 (45.5%)	0 (0%) ^#^
Hp^+^ X	25	0 (0.0%)	9 (36.0%) ^*#^	15 (60.0%) ^*^	1 (4.0%) ^#^
Hp^+^ G	27	0 (0.0%)	1 (3.7%)	16 (59.3%)	10 (37.0%)

Note: Data were analyzed by Kruskal–Wallis test followed by Bonferroni’s correction post-hoc test

^*^p < 0.05 VS. Hp^−^ X group; ^#^*p* < 0.05 VS. Hp^+^ G group

The Hp^+^ X group had a lower proportion of patients with mild inflammation compared to the Hp^−^ X group (*p* < 0.05), but a higher proportion with moderate inflammation (*p* < 0.05). There was no statistically significant difference in the proportion of patients in the Hp^−^ G group who developed all levels of chronic inflammation in the gastric mucosa compared with those in the Hp^−^ X group (*p* ≥ 0.05). In the Hp^+^ G group, the proportion of patients with mild inflammation was significantly lower than in the Hp^−^ G group (*p* < 0.05) and Hp^+^ X group (*p* < 0.05), while the proportion with severe inflammation was significantly higher than in the Hp^−^ G group (*p* < 0.05) and Hp^+^ X group (*p* < 0.05).

#### Gastric mucosal active inflammation was assessed in each group (Table 5), revealing a significantly lower incidence of inactive inflammation in the Hp^+^ X group compared to the Hp^−^ X group (*p* < 0.05)

**Table 5:** Grading of active inflammation of the gastric mucosa

Groups	*n*	Activity intensity			
		0	1	2	3
Hp^−^ X	26	22 (84.6%)	4 (15.4%)	0 (0.0%)	0 (0.0%)
Hp^−^ G	22	15 (68.2%) ^#^	5 (22.7%)	2 (9.1%) ^#^	0 (0.0%)
Hp^+^ X	25	2 (8.0%) ^*^	17 (68.0%) ^*#^	6 (24.0%) ^#^	0 (0.0%)
Hp^+^ G	27	0 (0.0%)	6 (22.2%)	19 (70.4%)	2 (7.4%)

Note: Data were analyzed by Kruskal–Wallis test followed by Bonferroni’s correction post-hoc test

^*^p < 0.05 VS. Hp^−^ X group; ^#^*p* < 0.05 VS. Hp^+^ G group

Conversely, the Hp^+^ X group exhibited a significantly higher rate of mild active inflammation compared to the Hp^−^ X group (*p* < 0.05). There was no statistically significant difference in the proportion of patients in the Hp^−^ G group who developed all levels of active inflammation in the gastric mucosa compared with those in the Hp^−^ X group (*p* ≥ 0.05). In the Hp^+^ G group, the prevalence of inactive inflammation was significantly lower than in the Hp^−^ G group (*p* < 0.05), while the rates of mild active inflammation were significantly lower than in the Hp^+^ X group (*p* < 0.05). Moreover, the incidence of moderate active inflammation was significantly higher in the Hp^+^ G group compared to both the Hp^−^ G and Hp^+^ X groups (*p* < 0.05).

### Effects of hypoxia on Hp-induced oxidative stress in gastric

**Fig. 2:**
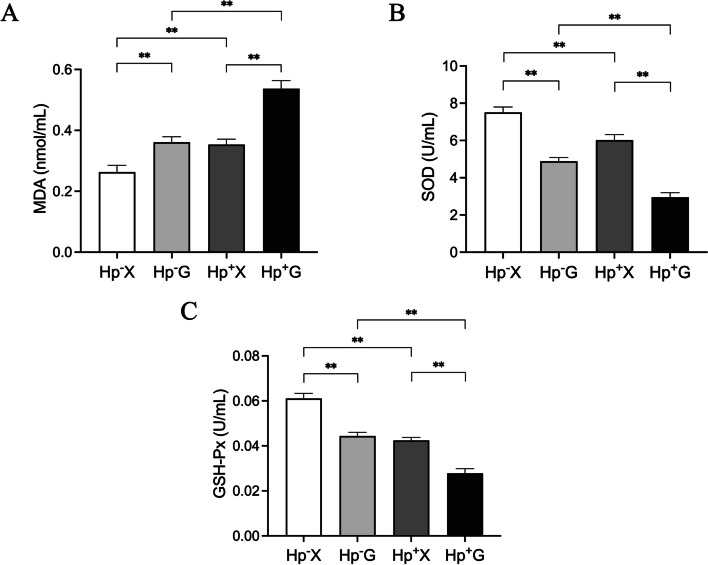
The levels of MDA (**A**), SOD (**B**), GSH-Px (**C**). Data were represented as mean ± SEM and analyzed by one-way ANOVA followed by LSD post-hoc test.^**^p < 0.01

Serum oxidative stress indexes were assessed in patients across different groups (Fig. 2), revealing that individuals in the Hp^+^ X group exhibited significantly elevated MDA levels compared to those in the Hp^−^ X group (*p* < 0.01), along with notably decreased SOD and GSH-Px activities (*p* < 0.01). Similarly, patients in the Hp^−^ G group demonstrated significantly higher MDA levels than those in the Hp^−^ X group (*p* < 0.01), coupled with decreased SOD and GSH-Px activities (*p* < 0.01). Furthermore, individuals in the Hp^+^ G group displayed significantly higher MDA levels than both the Hp^−^ G group (*p* < 0.01) and Hp^+^ X group (*p* < 0.01), with concomitantly lower SOD and GSH-Px activities compared to the Hp^−^ G group (*p* < 0.01) and Hp^+^ X group (*p* < 0.01). These findings suggest that hypoxia exposure exacerbates oxidative stress induced by Hp infection.

### Effects of hypoxia on Hp-induced inflammatory response in gastric

**Fig. 3:**
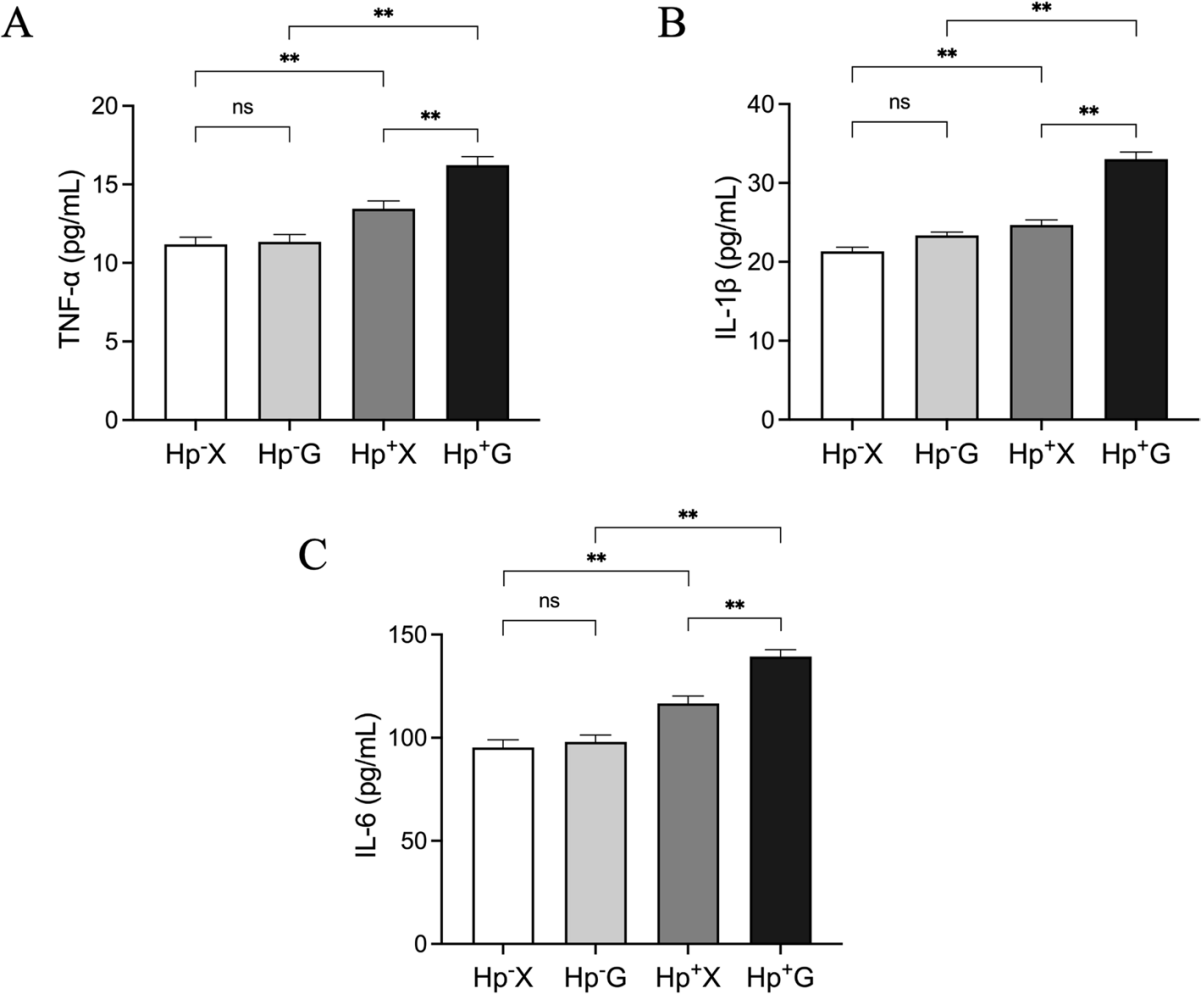
The levels of TNF-α (**A**), IL-1β (**B**), IL-6 (**C**). Data were represented as mean ± SEM and analyzed by one-way ANOVA followed by LSD post-hoc test. ^**^p < 0.01, ns, not significant

Serum inflammatory factors were assessed in patients across different groups (Fig. 3). The findings revealed significantly elevated levels of TNF-α, IL-1β, and IL-6 in patients from the Hp^+^ X group compared to those in the Hp^−^ X group (*p* < 0.01). Conversely, no statistically significant variances were observed in the levels of TNF-α, IL-1β, and IL-6 between patients in the Hp^−^ G and Hp^−^ X groups (*p* ≥ 0.05). Remarkably, patients in the Hp^+^ G group exhibited notably higher levels of TNF-α, IL-1β, and IL-6 compared to both the Hp^−^ G group (*p* < 0.01) and the Hp^+^ X group (*p* < 0.01). These results suggest that hypoxia exposure exacerbates the release of pro-inflammatory factors induced by Hp infection.

## Discussion

The diverse pathological manifestations of Hp infection stem from the intricate interplay of bacterial virulence, host genetics, and environmental influences, giving rise to various chronic gastritis phenotypes [8]. High-altitude environments are widely distributed globally, with an estimated 81.6 million people residing in regions above 2500 m [12]. A study investigating patients with digestive symptoms in high-altitude regions of India found a higher prevalence of Hp infection and gastritis severity through histopathology and endoscopy [13]. Furthermore, research indicates a notably elevated incidence of gastric perforation in young soldiers stationed in high-altitude areas, possibly linked to the high prevalence of gastritis and Hp infection among residents in these regions [14]. Therefore, the high incidence of Hp-related gastritis in high-altitude regions underscores the importance of studying the pathogenesis of Hp-related gastritis under hypoxic conditions for maintaining gastrointestinal health in high-altitude populations. Our study aims to investigate the impact of a high-altitude hypoxic environment on Hp infection-induced oxidative stress, inflammatory response, and gastric mucosal damage.

Chronic gastritis typically presents endoscopically with manifestations such as mucosal erythema, edema, and increased mucus, often accompanied by hemorrhagic dots or plaques and erosion [15]. Our study revealed a higher prevalence of diffuse erythema, enlarged folds, and white turbid mucus in the gastric mucosa of patients in the Hp^+^ X and Hp^+^ G groups compared to those in the Hp^−^ X and Hp^−^ G groups, respectively. Moreover, the Hp^+^ G group exhibited a significantly higher incidence of diffuse erythema and fold enlargement in the gastric mucosa compared to the Hp^+^ X group. These findings suggest that patients with Hp gastritis typically exhibit diffuse erythema, fold swelling, and white turbid mucus during endoscopy. In high-altitude, low-oxygen environments, the presence of Hp infection should be considered when observing diffuse erythema and fold enlargement in the gastric mucosa of patients with chronic gastritis.

Chronic gastritis is an inflammatory lesion of the gastric mucosa characterized by lymphocyte and plasma cell infiltration, and when accompanied by neutrophil infiltration it is called chronic active gastritis [16]. Our findings showed that patients in the Hp^+^ G group had the highest gastric mucosal inflammation score, suggesting that hypoxic exposure aggravates Hp gastritis. We further graded for the degree of chronic inflammation and the degree of active inflammation, and the results suggested that patients in the Hp^+^ G group had the lowest percentage of gastric mucosa with mild chronic inflammation and the highest percentage with severe chronic inflammation. In addition, patients in the Hp^+^ G group had the lowest rate of mild active inflammation and the highest rate of moderately active inflammation in the gastric mucosa. The above findings suggest that hypoxia exposure exacerbates the degree of chronic inflammation and active inflammation in the gastric mucosa of patients with Hp gastritis.

When Hp infects gastric mucosal epithelial cells, its type IV secretion system facilitates the delivery of virulence factors into the host cells [17]. Subsequently, these factors are recognized by pattern recognition receptors, triggering immunoinflammatory and oxidative stress signaling pathways [18, 19]. This activation leads to the release of pro-inflammatory cytokines and an accumulation of reactive oxygen species and reactive nitrogen species, ultimately causing gastric mucosal damage and exacerbating Hp-induced gastritis. Our findings indicate that individuals in the Hp^+^ G group exhibited elevated levels of serum TNF-α, IL-1β, IL-6, and MDA, along with reduced activities of serum SOD and GSH-Px. This suggests that hypoxic exposure intensifies the oxidative stress and inflammatory response provoked by Hp infection. Hypoxia-inducible factor-1α (HIF-1α), a widely distributed transcriptional activator in mammals and humans during hypoxia, is known to play a crucial role in the pathogenesis of various hypoxia-related diseases [20]. Although HIF-1α is typically absent in normal gastric mucosa, its expression intensifies progressively from Hp-induced gastritis to adenocarcinoma, indicating a potential molecular mechanism through which hypoxia exacerbates Hp infection-related gastric disorders [21].

The limitation of this study was that the sample size was relatively small, which may not be fully representative of the overall situation of patients with Hp-induced gastritis in high-altitude area. Future studies may consider conducting a large-scale, multicenter empirical study based on enlarging the sample size and considering the matching of samples between groups to verify the findings of this study.

## Conclusion

In conclusion, high-altitude hypoxia exacerbates gastric mucosal inflammation and injury by enhancing oxidative stress and inflammatory response induced by Hp infection. Therefore, increased emphasis on Hp detection and treatment is warranted for individuals residing in plateau regions to mitigate the prevalence and gravity of chronic gastritis. In addition, antioxidant therapy may be an effective strategy to alleviate Hp-associated gastritis conditions at high altitude. For this population, clinicians may consider antioxidants as an adjunctive therapy to mitigate gastric mucosal damage caused by oxidative stress and inflammation. This research offers novel insights for the prevention and management of chronic gastritis in plateau areas, enhancing comprehension and management of the impact of high-altitude hypoxia on Hp infection and associated conditions.

## Data Availability

The datasets used and/or analyzed during the current study are available from the corresponding author on reasonable request.

## Abbreviations

Hp: *Helicobacter pylori*
MDA: Malondialdehyde
SOD: Superoxide dismutase
GSH-Px: Glutathione peroxidase
TNF-α: Tumor necrosis factor-α
IL-1β: Interleukin-1β
IL-6: Interleukin-6
HIF-1α: Hypoxia-inducible factor-1α
LSD: Least significant difference
